# College Students’ Reduced Cognitive Empathy and Increased Anxiety and Depression before and during the COVID-19 Pandemic

**DOI:** 10.3390/ijerph191811330

**Published:** 2022-09-09

**Authors:** Janelle S. Peifer, Gita Taasoobshirazi

**Affiliations:** 1 Department of Psychology, University of Richmond, Richmond, VA 23173, USA; 2 School of Data Science and Analytics, Kennesaw State University, Kennesaw, GA 30144, USA

**Keywords:** anxiety, depression, intrapersonal identity, ethnic identity, cognitive empathy, college students, COVID-19, longitudinal data, pandemic

## Abstract

This study explored college students’ individual mental health (i.e., anxious and depressive symptoms, intrapersonal identity, and ethnic identity), as well as interpersonal mental health, as assessed by their affective connection to and care for others (i.e., cognitive empathy), exploring the role of culture and identity during the twin COVID-19 and racial justice pandemics of 2020. Comparing a longitudinal cohort of 147 undergraduate students’ experiences prior to the pandemic (Spring 2019) and after the onset of the pandemic (Spring 2021), the study examines students’ mental health changes amidst the multi-layered challenges of this time. A repeated measures Multivariate Analysis of Variance (MANOVA) revealed heightened anxiety and depression scores from pre-pandemic to during the pandemic and a reduction in cognitive empathy as expressed through perspective taking and empathic concern. The study begins to examine the implications of these findings in the COVID-19 era with a focus on young adult mental health, higher education, empathy, and community-mindedness.

## 1. Introduction and Literature Review

History has shown that global crises can impact individuals and their communities for generations [1]. The field of higher education and societies across the world will grapple with the academic, social, and emotional ripple effects of the COVID-19 pandemic and racial justice reckonings related to police brutality for decades to come. In particular, the simultaneous, “twin pandemics” of 2020 impacted students during a critical juncture of emerging adulthood and identity formation [2]. During this window of time, young adults reported heightened distress including dramatically heightened levels of anxiety, depressive symptoms, and suicidal ideation [3]. In addition, interpersonally and socially, students reported record levels of isolation and disconnection from others [4].

Prior to the pandemic, college student mental wellness had become a point of concern over the past 50 years, with mental health disorders making up over half of the disease morbidity for college-age adults in high-income countries [5]. Students’ emotional wellness is critical to their holistic success. Those with heightened distress also have a higher risk of early departure from their degree program [6,7], a lower sense of belonging and connection to their institution [8], and lower academic performance and achievement [9]. Additionally, individuals experiencing higher distress may be less likely to engage with others, report lower levels of empathy, and cite social impairments [10]. Taken together, the individual and social repercussions of college student mental health require attention—not only during the college years but also considering the implications for their post-graduate trajectories. As we understand how students’ psychosocial functioning has changed during the global pandemic, we can then elucidate the next steps for prevention and intervention initiatives.

The current study sought to explore the initial impact of the pandemic on college students’ mental health functioning individually (i.e., anxiety and depressive symptoms, identity, and ethnic identity) as well as interpersonally through their affective connection to and care for others (i.e., cognitive empathy). Given the nature of the pandemic, including the increased racial tension around police brutality, extrajudicial murder of George Floyd, and mainstream focus on the Black Lives Matter movement, we posit that students’ reflection on culture, identity, and race was heightened during these tumultuous years in ways that may have impacted their ethnic identity and intercultural competence. By comparing a longitudinal cohort of students’ experiences prior to the pandemic (Spring 2019) and after the onset of the pandemic (Spring 2021), we have the opportunity to examine how students’ mental health may have changed amidst the multi-layered challenges of 2020. Moreover, this study examines *intra*personal processes (anxiety and depression, identity and sense of self, ethnic identification) as well as *inter*personal ones (cognitive empathy).

## 2. Theoretical Framework

The symptoms of anxiety and depression include disturbances experienced both within an individual and his/her/their interaction with their social environment [11]. In one theory, self-focused attention (SFA) with a negative valence exacerbates depressive symptoms [12]. Those with anxious and depressive symptoms have heightened self-focused attention and negative affect [13]. Individuals with anxiety and depression tend to be attuned to perceived faults in themselves, ruminate more frequently, and have a heightened cognitive bias towards remembering mistakes and painful memories over a more holistic and balanced reflection [13]. As anxious and depressive symptoms mount, this can lead to ruminative worry and self-isolation. This, in turn, can create a feedback loop or self-fulfilling prophecy where anxious and depressive symptoms heighten alongside negative self-focused attention that then hinders social engagement and social support, which then can lead to more anxiety and depression [13]. One focus of the gold-standard evidence-based treatment for anxiety and depression—cognitive behavioral therapy (CBT)—can be to bolster a client’s awareness of this pattern to help adjust cognitive distortions and increase social support, engagement, and behavioral activation [14].

From a public health standpoint, it is crucial to understand how crises of this scale impact individuals and shape social variables. In the case of a viral pandemic, we recognize that empathy and community-mindedness are critical in prevention and intervention efforts. To reduce the spread of COVID-19, young adult college students must have empathy for others and be able to think and feel outside of their individual experiences and risk factors. In addition, cognitive empathy has been linked to prosocial outcomes including emotional regulation, mindfulness, and affective perspective-taking [15,16,17]. Understanding the interplay between anxiety/depression and cognitive empathy can help us explore effective interventions to help alleviate students’ mental health symptoms while bolstering their civic engagement and connections to and care for others.

In this project, we posit that self-focused attention can exacerbate social isolation and interrupt other-focused attention. In other words, more anxious and depressive symptoms hinder empathy towards others and cloud individuals’ abilities of perspective-taking and empathic concern. We propose that the pandemic will lead to college students experiencing greater anxiety and depressive symptoms. As they report higher levels of anxiety and depression, we also hypothesize that they will demonstrate less empathy towards others.

## 3. Methodology

### 3.1. Participants

The sample included 147 undergraduate students from a diverse, private, small liberal arts college in the Atlanta metropolitan area in the United States that enrolls all genders, except cis-gender men. The demographic make-up of the sample is included in Table 1.

### 3.2. Procedures

This study used a within-subjects design comparing pre-pandemic (Spring 2019) and pandemic era data (Spring 2021) collected as part of a larger study: The Global Pathways Study (GPS). Both the Spring 2019 and Spring 2021 survey response rates were 25%. The GPS is a multi-institutional, longitudinal study examining college student development with an intercultural competence focus. Students were contacted with a link to complete the survey using Qualtrics online survey software. Students received an initial invitation via their college email address and received two follow-up reminders to complete the survey. The survey questionnaire took an average of 15 min to complete. Informed consent was collected from participants, and the study was conducted in compliance with the first author’s Institutional Review Board.

### 3.3. Measures

#### 3.3.1. Anxiety and Depression

Anxiety and depression were measured using the Patient Health Questionnaire, with 4 Items (PHQ-4; [18]). This ultra-brief measure has demonstrated validity and reliability for detecting symptoms of anxious and depressive distress with the first two items focused on anxiety (e.g., “feeling nervous, anxious, or on edge”) and the second two focused on depressive symptoms (e.g., “little interest or pleasure in doing things”). The response scale is a Likert scale (0 = not at all, 1 = several days, 2 = more than half the days, 3 = nearly every day). Cronbach’s alpha was 0.90 at pre-testing and 0.90 at post-testing.

#### 3.3.2. Cognitive Empathy

Cognitive empathy was assessed using two subscales (perspective-taking and empathic concern) from Davis’ (1980) Interpersonal Reactivity Index (IRI). The 28-item measure assesses an individual’s self-reported empathy to the observed experience of another [19] on a 5–point Likert scale where participants rate their agreement with how statements describe them ranging from “does not describe me well” to “describes me very well.” The 7-item perspective-taking subscale examines one’s tendency to take the psychological viewpoint and perspective of others. It includes items such as “I try to look at everybody’s side of a disagreement before I make a decision,” and “I believe that there are two sides to every question and try to look at them both.”

The 7–item empathic concern subscale assesses how participants feel sympathy and concern for others. Sample items such as “I often have tender, concerned feelings for people less fortunate than me,” and “I am often quite touched by things that I see happen” seek to capture the empathetic, emotional reaction of an individual to another’s subjective experience. Both subscales were combined, and composite scores were used to assess cognitive empathy. The overall cognitive empathy reliability was good with a Cronbach’s alpha of 0.72 for the sample at pre-testing and 0.78 at post-testing [19].

#### 3.3.3. Intercultural Competence: Intrapersonal Identity

Intercultural competence was measured using the New Student Form version of the Global Perspectives Inventory (GPI) [20]. This study utilized items from the intrapersonal identity subscale. In the intrapersonal dimension, the identity subscales assess the “level of awareness of one’s unique identity, sense of purpose, and degree of acceptance of one’s identity”. 

Students responded to 35 items using a 5-point Likert scale (1 = strongly disagree, 2 = disagree, 3 = neutral, 4 = agree, and 5 = strongly agree) on questions related to their own cultural identity and feelings towards those who are culturally different. Sample items included “Some people have a culture and others do not”, “I see myself as a global citizen”, and “I frequently interact with people from a different race/ethnic group than my own”. The overall scale had good reliability for this sample at pre-testing (ɑ = 0.70) and post-testing (ɑ = 0.81).

#### 3.3.4. Ethnic Identity

Ethnic identity was assessed using Brown et al.’s (2014) Multigroup Ethnic Identity Measure-Revised (MEIM-R; [21]) revised from Phinney’s Multigroup Ethnic Identity Measure [22]. The MEIM-R measures participants’ self-reported affiliation with one’s ethnic group across two dimensions: (1) Commitment (three items): One’s sense of belonging to an identity, and (2) exploration (three items): Exploring the meaning of one’s identity. The items are on a five-point Likert scale ranging from (1) Strongly Disagree to (5) Strongly Agree. For this sample, internal consistency, as measured by Cronbach’s alpha, was 0.80 for pre-testing and 0.77 for post-testing.

## 4. Data Analysis

All analyses were run using SAS 9.4, including descriptive statistics and multivariate analyses. An a priori power analysis using G*Power confirmed that our sample size of 147 was sufficient for a repeated measures Multivariate Analysis of Variance (MANOVA) with six dependent variables (with an alpha of 0.05, power of 0.80, an effect size of f = 0.25, and the recommended sample size is *n* = 42). The data met the assumptions of a repeated measures MANOVA including independent and continuous observations, multivariate normality, and no multicollinearity. Missing data (less than 2% of the total data) were eliminated using listwise deletion.

## 5. Results

Means and standard deviations are reported in Table 2. The values in Table 2 show the means and standard deviations from pre to post and the values in bold highlight significant changes from pre to post. Results from the repeated measures MANOVA suggested a significant effect of time, Wilks’ Lambda = 0.774, F (5, 142) = 8.30, *p* < 0.001, partial eta squared = 0.23. From among the dependent variables, there was a significant decrease in cognitive empathy from pre- to post-testing, F = 35.43, *p* < 0.001, partial eta squared = 0.20. In addition, there was a marginally significant increase in anxiety from pre- to post-testing, F = 3.46, *p* = 0.06, partial eta squared = 0.02. When gender or race was included in the model with time, there was no significant interaction or main effect for the demographic variables.

## 6. Discussion and Implications

This longitudinal design of the study enabled us to examine changes within individuals, analyzing shifts that occurred before the onset of the pandemic (Spring 2019) to the pandemic era (Spring 2021). Moreover, we examined culture, race, and identity variables, recognizing the differential impact that the pandemic has had on minoritized and historically marginalized groups.

Findings from this study align with research that college students and young adults’ mental health declined during the pandemic [23,24]. Our results indicated an increase in anxiety and depression scores from pre- to post-test. These findings may be influenced by the stress and isolation experienced during the pandemic. Research has indicated that college students experienced particular strain due to the disruption to their social networks [25,26] and access to on-campus mental health resources [27]. It is possible that anxiety and depression scores rose over the two-year period due to other factors, unrelated to the pandemic. Further qualitative research would need to query students about factors that impacted their responses to these questions to isolate the variables most influential on mental health. Understanding the changes in anxiety and depression can help equip colleges and universities to provide additional resources, psychoeducation, and identification and support to students who may have heightened and exacerbated mental health concerns in the pandemic era and its eventual wake.

We were surprised to observe that intercultural competence and ethnic identity did not change during the twin pandemic. Future research can further elucidate why this may be, but this may be attributable to the timing of the surveys. The survey timing preceded the heightened racial tension that came to a boiling point during Summer 2020 with an increased focus on the Black Lives Matter movement. While shifts in identity likely occurred during these times, the measure may not have been timely or sensitive enough to fully capture the shifts that students may have experienced in their ethnic identity and sense of self.

In addition, we found a reduction in cognitive empathy from pre- to post-pandemic. This aligned with our theoretically grounded hypotheses that increased stressors and distress may heighten self-focused attention and social isolation, while hindering empathic attunement towards others. Of course, other factors may have influenced the empathy scores—the timing of the pandemic coincided with long periods of mandated and voluntary social isolation and political polarization. At the same time, the stress of the pandemic may have had the somewhat counterintuitive impact of heightening self-focus and lowering empathy towards others. During a period of massive and pervasive stress with suffering on an almost inconceivable level, individuals may focus more on their own needs and survival. This possibility is vital for higher education institutions to attend to and be aware of in their work.

One limitation of the present study is that only two time points were examined. We recommend follow-up analyses of students, now two years after the beginning of the pandemic, to evaluate their empathy, self-efficacy, anxiety and depression, intercultural competence, and ethnic identity. A second limitation is that the current study is a single-gender-sample study. Future research should include additional genders to improve generalizability.

## 7. Conclusions

Given that many institutions prioritize services, citizenship, and social impact beyond the individual benefits that higher education can bestow upon its students, the rebound from the pandemic era may be beneficial at both the individual (e.g., bolstering mental health services, debriefing the pandemic trauma, modifying academic policies) and social levels (e.g., providing scaffolding for social network building, encouraging empathy and citizenship, community-based learning courses). Our findings highlight the importance of institutions, policy-makers, and individuals foregrounding mental health and connection and care for others simultaneously. As the world continues to grapple with the far-reaching implications of a devastating, global trauma, higher education institutions are well-positioned to provide wrap-around care to individuals while educating them to develop empathic attunement and connection to respond to the challenges of our time.

## Figures and Tables

**Table 1:** Demographics.

Demographics	
Race 64 (44%) White/Caucasian 44 (30%) Black/African American 13 (9%) Latino(a)(x)/Hispanic 11 (7%) Asian 1 (0.6%) Pacific Islander 14 (9%) Other	Gender Identity 119 (81%) Female 14 (10%) Transgender Male 6 (4%) Male 4 (3%) Unsure 1 (0.6%) Gender Fluid 1 (0.6%) Transgender Female 1 (0.6%) Prefer Not to Say 1 (0.6%) Not Listed Here

**Table 2:** Descriptive statistics.

**Dependent Variable**	**Pre (*n* = 147)**	**Post (*n* = 147)**
**Anxiety/Depression**	**M = 9.35, SD = 3.79**	M = 10.14, SD = 3.75 *
**Cognitive Empathy**	**M = 45.78, SD = 4.87**	M = 42.35, SD = 4.25 **
Intrapersonal Identity	M = 23.85, SD = 2.89	M = 23.49, SD = 3.76
Ethnic Identity	M = 17.57, SD = 3.44	M = 17.77, SD = 3.25

Note. * *p* < 0.05, ** *p* < 0.01. Bolded variables and accompanying statistics are significant.

## Data Availability

Data, methods used in the analysis, and materials used to conduct the research will be made available to any researcher for purposes of reproducing the results or replicating the procedure. To do so, submit a request to the author by e-mail.
